# Population genetic structure of the malaria vector *Anopheles funestus*, in a recently re-colonized area of the Senegal River basin and human-induced environmental changes

**DOI:** 10.1186/1756-3305-5-188

**Published:** 2012-09-05

**Authors:** Badara Samb, Ibrahima Dia, Lassana Konate, Didier Fontenille, Anna Cohuet

**Affiliations:** 1 Département de Biologie animale Laboratoire d’écologie vectorielle et parasitaire, Université Cheikh Anta Diop de Dakar, Dakar-Fann, BP 5005, Sénégal; 2 Unité d’entomologie médicale, Institut Pasteur de Dakar, 36 Avenue Pasteur, Dakar, BP 220, Sénégal; 3 Institut de Recherche pour le Développement, Unité MIVEGEC (IRD 224-CNRS 5290-UM1-UM2), 34394, Montpellier Cedex 5, BP 64501, France; 4 Institut de Recherche en Sciences de la Sante’-Direction Régionale de l’Ouest, Bobo Dioulasso, Burkina Faso; 5 Department of Biological Sciences Eck Institute for Global Health, University of Notre Dame, Notre Dame, IN, 46556, USA

**Keywords:** *Anopheles funestus*, Re-emergent population, Population structure, Microsatellites, Senegal river basin

## Abstract

**Background:**

*Anopheles funestus* is one of the major malaria vectors in tropical Africa. Because of several cycles of drought events that occurred during the 1970s, this species had disappeared from many parts of sahelian Africa, including the Senegal River basin. However, this zone has been re-colonized during the last decade by *An. funestus,* following the implementation of two dams on the Senegal River. Previous studies in that area revealed heterogeneity at the biological and chromosomal level among these recent populations.

**Methods:**

Here, we studied the genetic structure of the newly established mosquito populations using eleven microsatellite markers in four villages of the Senegal River basin and compared it to another *An. funestus* population located in the sudanian domain.

**Results:**

Our results presume Hardy Weinberg equilibrium in each *An. funestus* population, suggesting a situation of panmixia. Moreover, no signal from bottleneck or population expansion was detected across populations. The tests of genetic differentiation between sites revealed a slight but significant division into three distinct genetic entities. Genetic distance between populations from the Senegal River basin and sudanian domain was correlated to geographical distance. In contrast, sub-division into the Senegal River basin was not correlated to geographic distance, rather to local adaptation.

**Conclusions:**

The high genetic diversity among populations from Senegal River basin coupled with no evidence of bottleneck and with a gene flow with southern population suggests that the re-colonization was likely carried out by a massive and repeated stepping-stone dispersion starting from the neighboring areas where *An. funestus* endured.

## Background

*Anopheles funestus* Giles, 1900 is one of the major malaria vectors in tropical Africa with *An. gambiae sensu stricto* and *An. arabiensis*[1], being the primary malaria vector in some areas [2-5]. In Senegal, *An. funestus* has been already described in all bio-geographic zones [6] and exhibits a predominant role in malaria transmission [7-9]. Following the recurrent droughts that have occurred during the 1970s, this species had disappeared from many parts of sahelian Africa, including the Senegal River basin, in consequence of the disappearance of its specific breeding sites [10]. However, after more than three decades of absence, the re-emergence of *An. funestus* was reported at the beginning of this century in the low valley of the Senegal River [11]. The hydro-agricultural implementations following the start-up of the Diama dam are highly suspected to create favourable breeding places for the re-establishment of *An. funestus*[9,12]. This re-colonization had thus given rise to the fear of recrudescence of the transmission and incidence of malaria in this area. An entomological survey carried out thereafter in the Senegal River basin showed heterogeneity of *An. funestus* populations in their anthropophily, densities and parity [9]. Because this species is known to be biologically and genetically highly polymorphic [13-17], these observations led us to suspect a potential genetic structure within the newly established *An. funestus* populations. Likewise, significant chromosomal differentiation not linked to geographical distance has been reported between the *An. funestus* populations from the Senegal River basin and those from the sudanian domain [12].

In the present study, we studied the genetic structure of the *An. funestus* populations in the Senegal River basin using microsatellite DNA markers. This set of molecular markers were demonstrated to be suitable tools for population genetics studies within this mosquito [17-20]. We aimed at investigating the genetic diversity and the genetic structure of the newly established *An. funestus* populations in the Senegal River basin in comparison to a more southern population of this species.

## Methods

### Study sites and mosquito collection

**Figure 1:**
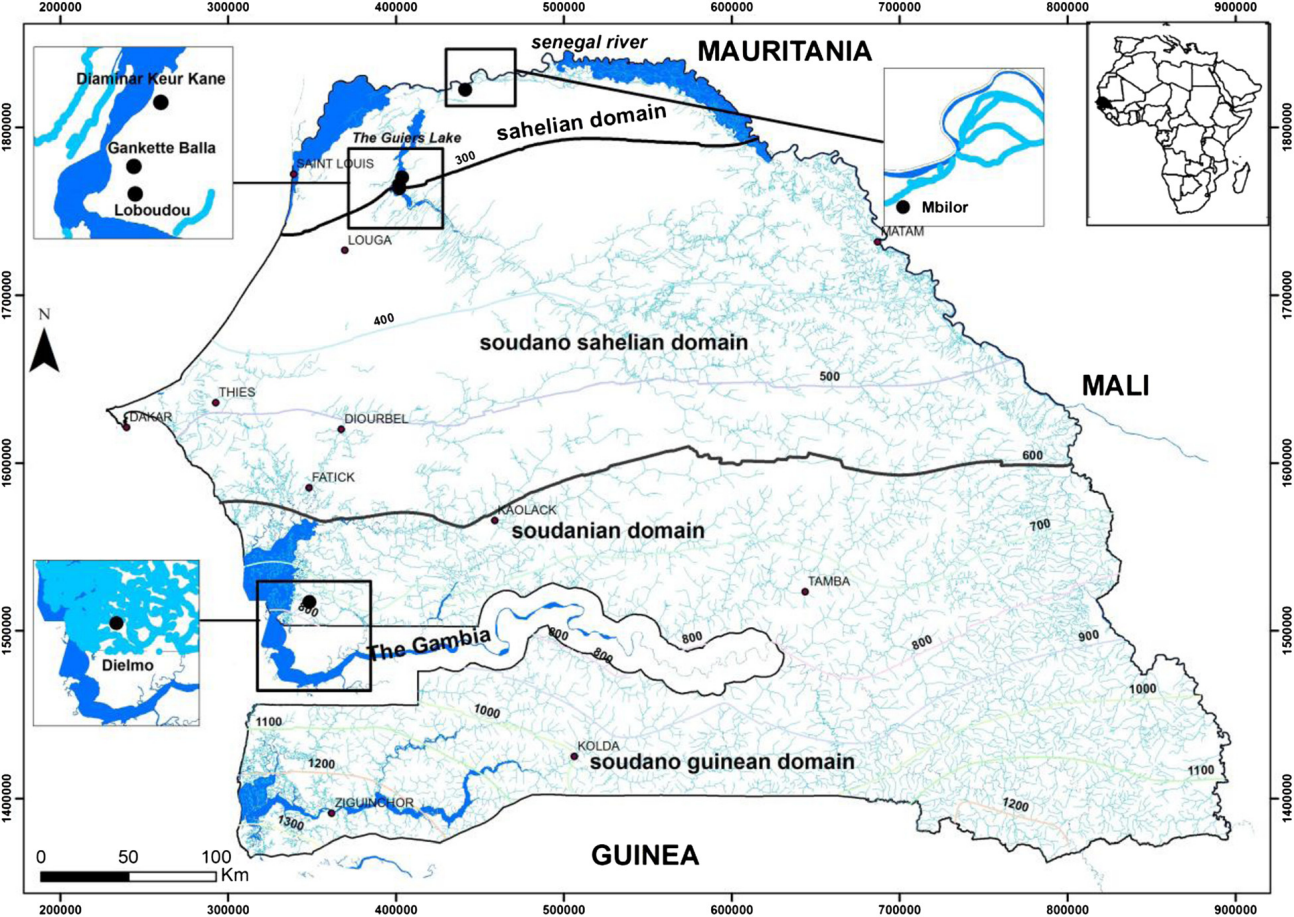
**Map of Senegal showing collection sites of *An. funestus *from four villages located in the Senegal River Basin, in Sahelian domain and from the village of Dielmo, located in the Sudanian domain.**

The study was carried out in four villages located in the Senegal River Basin, in sahelian domain (Mbilor, Gankette Balla, Diaminar Keur Kane, Loboudou) and in the village of Dielmo, located in the sudanian domain (Figure 1). The re-emergence of *An. funestus* was previously observed in the four selected villages from the Senegal River basin and biological heterogeneities were observed between sites [9]. The village of Mbilor (16°29' N, 15°33' W) is situated in the low valley of the Senegal River. A retention basin made by the Senegalese Sugarcane Company and derived from the Senegal River represents the unique source of permanent water, which represents the main breeding site of anopheline mosquitoes. The village of Gankette Balla (15°58'N, 15°55'W), Diaminar Keur Kane (16°00'N, 15°54'W) and Loboudou (15°57'N, 15°55'W) are situated on the shores of The Guiers Lake. The association between the shores of the Guiers Lake and the development of fresh water plants represent the main breeding sites for *An. funestus,* being the predominant anopheline species collected [9]. Mosquito collections were carried out between October 2003 and September 2004 in these four villages.

Mosquitoes from the Senegal River basin populations were compared to Dielmo (13°45’N, 16°25’W), located in a sudanian domain of central Senegal, on the marshy bank of a small permanent stream, where anopheline mosquitoes breed all year round. Specimens from Dielmo were collected during the rainy season in 2000, between July and October.

All mosquitoes were collected by indoor pyrethrum spraying in randomly selected human dwellings. After collection, *An. funestus* were identified morphologically [21] and stored individually in labelled tubes with desiccant for further molecular processing.

### DNA extraction, molecular species identification and microsatellite genotyping

Genomic DNA was extracted from wings and legs of each individual mosquito following the protocol described by Morlais *et al.*[22]. DNA was then resuspended in sterile water in individual tubes. Morphological identification of *An. funestus sensu stricto* was confirmed by molecular methods [23,24]. Eleven microsatellite loci were selected from published *An. funestus* sequence data [25-28], based on their high polymorphism and no evidence for null alleles. These microsatellites were *FunL, FunO, AFUB11, AFND19, AFND20, AFND32, FunQ, AFND40, AFND5, FunG* and *FunF.*

PCR was performed for each locus individually in a 25 μl reaction volume. The reaction mixture contained 1 X PCR buffer containing 1.5 mM MgCl_2_ (Promega, France), 0.2 mM of each dNTP (Eurogentec, Belgium), 10 pmol of each primer, 0.1U of Taq DNA polymerase (Promega, France) and approximately 5–10 ng of template DNA. The forward primer was labelled in 5’ with either by Fam, Vic, Ned or Pet fluorescent markers to allow multiplex electrophoresis (Eurogentec, Belgium). Amplification was performed in a GeneAmp 9700 thermocycler (Applied Biosystems, France). Cycling conditions were: an initial denaturation step at 94°C for 2 min followed by 36 cycles of 30 s at 94°C, 30 s at 54°C, 30 s at 72°C and a final elongation step of 10 min at 72°C. Amplified products were diluted and pooled for genotyping in AB3130 sequencer (Applied Biosystems, France). Alleles were sized relatively to an internal size standard using GENEMAPPER v4.0 (Applied Biosystems, France).

### Data analysis

For each locus and each population sample, heterozygosity was computed using GENETIX v.4.05 [29] and the number of alleles were computed using FSTAT v.2.9.3.2 [30]. FSTAT and GENEPOP v.4.0.3 [31] were used to assess the deviation from Hardy-Weinberg equilibrium at each locus, each population sample, and overall as indicated by the inbreeding coefficient (*F*_*IS*_*)*. Linkage disequilibrium between pairs of microsatellite loci was assessed using FSTAT v.2.9.3.2 [30]. Significance was tested using the randomization approach implemented in FSTAT with Bonferroni-adjusted *P*-values.

Genetic differentiation between populations was assessed by estimating Wright's *F*-statistics [32], calculated according to Weir & Cockerham [33]. Statistical significance of *F*_*ST*_ was assessed using G-based exact tests for genotypic differentiation [34], available in GENEPOP.

Because demographic instability such as recent population bottleneck and/or expansion might bias genetic differentiation, heterozygosity tests were implemented to test for Mutation-Drift Equilibrium (MDE) using BOTTLENECK V1.2.02 [35]. At selectively neutral loci, the expected heterozygosities calculated from allele frequency data (*He*) and from the number of alleles and sample sizes (*Heq*) are expected not to be significantly different (*H*e ≈ *H*eq) in a population at MDE. If a population experiences a bottleneck, rare alleles will be lost by genetic drift, and *H*eq will decrease faster than *H*e (*H*e > *H*eq). The resulting apparent excess of heterozygotes is an indicator of a recent bottleneck event, while the opposite (*H*e < *H*eq) may suggest population expansion. Estimates of expected heterozygosity under MDE were calculated using two mutation models for microsatellite evolution: the Two Phased Mutation model (TPM) [36] and the Stepwise Mutation Model (SMM) [37]. Wilcoxon signed rank tests were used to determine whether deviations from MDE were statistically significant.

We applied a Bayesian model-based clustering algorithm to infer population structure and to assign individuals (probabilistically) to clusters without a priori knowledge of population units and limits. This approach, implemented in STRUCTURE v2.2 [38], uses individual multilocus genotype data to cluster individuals into *K* groups while minimising Hardy-Weinberg disequilibrium and gametic phase disequilibrium between loci within groups [39]. In STRUCTURE v 2.2, the number of distinct genetic clusters in the data set (K) was estimated from 1 to 12 by the posterior log probability of data under each K, Ln [Pr(X|*K*)] [38]. Each run carried out 500,000 iterations after a burn-in period of 40,000, using the admixture model and correlated allele frequencies. To check for convergence of the Markov chain Monte Carlo (MCMC), we performed over 5 replicates for each value of *K* and then checked the consistency of results [40]. The estimated number of clusters (*K*) was taken to be the value of *K* with the highest Pr (X|*K*) [39].

Groups of populations were defined according to STRUCTURE results and the hierarchical genetic variation existing within groups of populations and within populations was analyzed by Analysis of Molecular Variance (AMOVA) using Arlequin version 3.5 [41].

The correlation between genetic and geographical distances was assessed by the regression of *F*_*ST*_/(1 – *F*_*ST*_) on the logarithm (ln) of geographical distance [42], and tested using the Mantel test available in GENEPOP. Dispersion ability was estimated by the “neighborhood size,” Dσ2, that is, the product of the effective population density (D) by the average squared parent offspring distance (σ^2^) [42].

Kruskal-Wallis test was used to compare the average number of alleles and the average proportions of heterozygosity between populations using XLSAT Pro 2006®.

The Bonferroni correction procedure [43] was applied to evaluate significance when multiple tests were performed.

## Results

### Genetic variability

Genotypes of 235 *An. funestus* females were scored at 11 microsatellite loci. These microsatellite loci were highly polymorphic with a number of distinct alleles per locus ranging from 6 (*FUNQ, AFND5*) to 15 (*AFND32, FUNL*) in our five populations (Table 1). The average number of alleles per locus ranged from 6.8 to 7.7 and was not significantly different among populations (*P* = 0.83). Mean observed heterozygosity across all loci ranged from 0.68 to 0.72 and was not significantly different among populations (*P* = 0.89). Heterozygosity tests were performed to explore demographic stability in *An. funestus* populations and compliance to mutation-drift equilibrium. Significant deviations from mutation-drift equilibrium, as a result of heterozygote excess, was found in a population of Loboudou, when the studies were performed under TPM 70% and TPM 90% model. In Dielmo, significant deviations associated with strong heterozygote deficiencies were detected under SMM model. For the populations of Gankette, Diaminar and Mbilor, no deviation from MDE was found (Table 2).

**Table 1:** **Genetic Variability at 11 microsatellites loci in *Anopheles funestus *from Senegal**

**Locus (chromosomal location)**		**Populations**					
		**Diaminar**	**Dielmo**	**Gankette**	**Loboudou**	**Mbilor**	**Overall**
		(N = 48)	(N = 41)	(N = 49)	(N = 47)	(N = 50)	(N = 235)
AFUB11	Nall	7	9	7	6	7	9
(2 L)	HO	0.5682	0.7576	0.5833	0.6383	0.4681	0.594
	F*is*	0.215	−0.009	0.106	0.046	**0.347**	**0.1530**
FUNL	Nall	10	11	7	9	9	15
(2 L)	HO	0.8864	0.7813	0.8140	0.6444	0.9375	0.816
	F*is*	−0.076	0.043	0.013	0.191	−0.094	0.0175
FUNO	Nall	9	7	8	7	8	10
(2R)	HO	0.7381	0.8125	0.7083	0.8085	0.8298	0.778
	F*is*	−0.010	−0.062	−0.036	−0.114	−0.102	−0.0363
AFND19	Nall	11	8	11	11	9	13
(3R)	HO	0.5870	0.7179	0.7234	0.7179	0.7200	0.692
	F*is*	**0.305**	0.010	0.137	0.127	0.141	**0.1673**
AFND20	Nall	8	8	7	8	6	10
(3R)	HO	0.7500	0.7949	0.7234	0.7500	0.6800	0.736
	F*is*	−0.045	−0.062	0.049	0.052	0.059	0.0232
AFND32	Nall	10	10	9	8	9	15
(2R)	HO	0.7609	0.7750	0.8723	0.8974	0.7000	0.797
	F*is*	0.101	0.081	−0.049	−0.073	0.102	0.0535
FUNQ	Nall	3	4	4	5	4	6
(X)	HO	0.5000	0.6500	0.4681	0.5128	0.6600	0.559
	F*is*	0.109	0.081	0.156	0.184	−0.021	**0.1162**
AFND40	Nall	6	7	7	7	5	8
(2R)	HO	0.8889	0.7250	0.8000	0.7273	0.7292	0.775
	F*is*	−0.146	−0.022	−0.051	0.085	−0.001	−0.0182
AFND5	Nall	5	6	5	5	5	6
(2R)	HO	0.3913	0.5000	0.5217	0.4783	0.4286	0.463
	F*is*	0.246	0.141	0.039	0.211	0.089	0.1493
FUNF	Nall	5	7	4	6	5	7
(3 L)	HO	0.7391	0.7500	0.7174	0.6739	0.6122	0.696
	F*is*	−0.138	0.028	−0.114	0.020	−0.067	−0.0285
FUNG	Nall	10	8	9	9	8	12
(3R)	HO	0.8043	0.7500	0.8261	0.7826	0.7917	0.792
	F*is*	0.063	−0.020	0.020	0.078	0.070	0.0854
Mean	Nall	7.6	7.7	7.09	7.3	6.8	10
across all loci	HO	0.6922	0.7286	0.7053	0.6938	0.6870	0.700
	F*is*	0.054	0.017	0.023	0.070	0.048	**0.06**

**Table 2:** **Heterozygosity tests in *An. funestus *populations from Senegal **

**Locality**	**TPM**			**SMM**
	**70%**	**80%**	**90%**	
Diaminar	3	4	4	7
Dielmo	7	7	7	10**
Gankette	3	3	4	7
Loboudou	1**	3*	7	8
Mbilor	2	5	5	6

**TPM**: Two-Phase mutation Model. **SMM**: Stepwise Mutation Model.

*P < 0.05, **P < 0.01 (two-tailed Wilcoxon signed-rank test P-values for deviation from MDE) after correction for multiple tests. The number of microsatellite loci showing heterozygote deficiency (e.g., He < Heq) out of 11 test loci is given.

### Hardy-Weinberg and linkage disequilibrium

When the pooled samples were analyzed as a single population, 3 (*AFUB11, AFND19* and *FUNQ*) of 11 loci showed significant deviations from Hardy-Weinberg equilibrium due to significant heterozygote deficiency (Table 1). When considering all loci, no deviation from Hardy-Weinberg equilibrium was observed within each population studied. However, significant deviation from Hardy-Weinberg expectations was observed for loci *AFUB11* and *AFND19* respectively in populations of Mbilor and Diaminar after the Bonferroni correction was applied. These deviations from Hardy-Weinberg expectations were associated with heterozygote deficiency (Table 1). No linkage disequilibrium was observed in any pair of loci (6600 permutations, *P* > 0.05).

### Genetic differentiation and structure analysis

The levels of genetic differentiation between pairs of populations were estimated by *F*_*ST*_ values. Table 3 shows *F*_*ST*_ estimates for all pairwise population comparisons. The values of *F*_*ST*_ between pairwise population comparisons for all loci ranged from 0 (Diaminar-Gankette) to 0.0519 (Dielmo-Mbilor). The highest *F*_*ST*_ estimates were obtained between the most distant populations. *F*_*ST*_ estimates were highly significant (*P* <10^-4^) between Dielmo and the populations of the Senegal River basin and between Mbilor and the populations around Guiers Lake area (Diaminar, Gankette and Loboudou). After all, only three comparisons were not significant after Bonferroni correction: Diaminar-Loboudou (*P* > 0.005), Diaminar-Gankette (*P* > 0.005), and Gankette-Loboudou (*P* > 0.005).

**Table 3:** **Estimates of *F***_***ST***_**values and their statistical significance (Bolded values)**

**Populations**	**Diaminar**	**Dielmo**	**Gankette**	**Loboudou**
Diaminar		--	--	--
Dielmo	**0.0463**		--	--
Gankette	−0.0004	**0.0490**		--
Loboudou	−0.0030	**0.0429**	0.0055	
Mbilor	**0.0092**	**0.0519**	**0.0125**	**0.0140**

*P*- values obtained after 10.000 permutations; Indicative adjusted nominal level (5%) for multiple (10) comparisons is 0.005.

*P* < 0.0001 for Bolded values.

**Figure 2:**
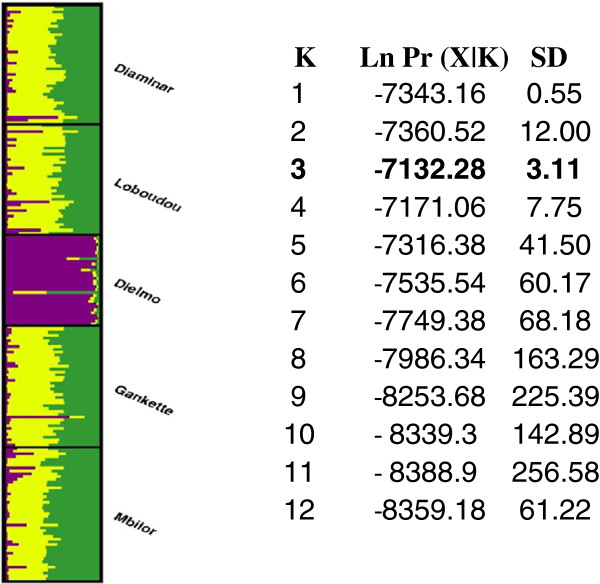
**Estimated population structure from Structure analyses: Mean (± SD) posterior probability variation of the data [LnPr(X|*K*)] (over 5 *Structure*replicated runs) between successive number of clusters (K) estimated using STRUCTURE v 2.2.**

*Structure v 2.2.* provided consistent results over 5 replicated runs tested for each *K*. The probability of the data (LnPr(X|*K*)) greatly increased from *K* = 1 to *K* = 2, and then reached a maximum value at *K* = 3, after which the values decreased gradually (Figure 2). Thus, in agreement with results based on *F*_*ST*_, the most likely number of genetic clusters in the dataset is *K* = 3 (Figure 2).

The analysis of molecular variance (AMOVA) of all the eleven microsatellites then confirmed the differentiation and structure analysis with the variation within individuals, among individuals within populations, among populations within groups and among groups being 92.73%, 4.21%, 0.08%, and 2.97% respectively (Table 4). AMOVA showed that the variation among populations within groups explained only 0.06% of the total variance while the variation from among individuals within populations and within individuals explained 4.21% and 92.73% of the total variation, respectively.

**Table 4:** **Analysis of molecular variance (AMOVA) of 11 microsatellite loci in the *An. funestus *populations from Senegal, for 5 populations and 3 groups defined as (Dielmo/Mbilor/Diaminar, Gankette and Loboudou)**

**Source of variation**	**Sum of squares**	**Variance components**	**Percentage variation**
Among groups	39.166	0.12333	2.97159
Among populations within groups	8.986	0.00339	0.08157
Among individuals within populations	907.273	0.17487	4.21357
Within individuals	850.500	3.84868	92.73328
Total	1805.926	4.15026	

### Isolation by distance

**Figure 3:**
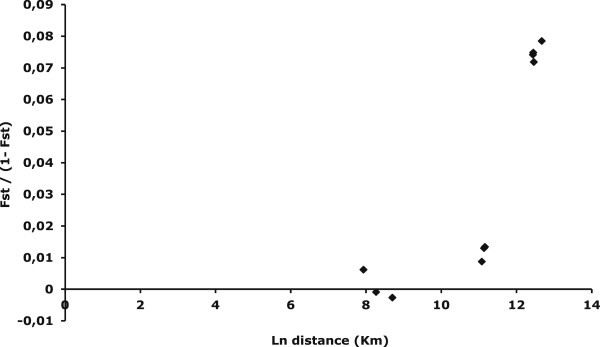
**Correlation between *F***_**ST**_**/(1-*F***_**ST**_**) and logarithm of distance (in Km) for pair-wise comparisons of 5 *Anopheles funestus *populations from Senegal genotyped at 11 microsatellite loci.**

Isolation by distance was tested and showed a positive and significant (R^2^ = 0.76, *P* = 0.02) correlation between *F*_*ST*_/(1- *F*_*ST*_) and the logarithm of distance, when considering the five villages (Dσ^2^ = 7.23) (Figure 3). The correlation became non significant when considering only the *An. funestus* populations of the Senegal River basin (R^2^ = 0.69, *P* = 0.20) (Dσ^2^ = 20.94).

## Discussion

This study revealed genetic stability among the populations of *Anopheles funestus* in the Senegal River basin. Moreover, our results showed high levels of genetic diversity within the re-emergent populations of the Senegal River basin and a permanent population in the Sudanian domain. The comparable levels of genetic diversity between both areas confirmed the genetic stability of the newly established populations from the Senegal River basin. Furthermore, geographical distance seems to be the key factor for population genetic divergence, although, other factors could potentially play a role in the genetic differentiation among Senegal River basin populations. The high levels of gene flow denote important mosquito migration among populations.

The observed genetic stability of *An. funestus* populations from the Senegal River basin was at least unexpected. Indeed, after several decades of periodic droughts, and recent re-emergence of *An. funestus*, we predicted a genetic signal of bottleneck followed by population expansion, as reported elsewhere in *An. gambiae*[44]. The absence of such demographic signals may be explained by the noteworthy effective population size of the *An. funestus* population, which could promptly foster population equilibrium [45]. For instance, the large population size in *Anopheles gambiae* prevented any impact on the mosquito population structure after strong population selection (i.e. vector control measures, dry seasons) [46,47]. On the other hand, high migration rates between populations can also erase any genetic signal of natural selection [48]. Therefore, the absence of signal for bottleneck and demographic expansion may indicate that the re-colonization was massive and/or seasonally repeated. We then hypothesize that the gene flow between Senegal River basin and other *An. funestus* populations may occur each year at the rainy season, when breeding sites are numerous and close enough to allow mosquito dispersal among populations.

Our results showed low but significant levels of genetic differentiation between populations of the Senegal River basin and those from the sudanian area. High levels of gene flow have been repeatedly reported on *An. funestus* and our estimates are consistent with previous studies on genetic structure of *An. funestus* populations in Senegal and other parts of Africa using microsatellites [17,18,20,49,50]. The important gene flow between *An. funestus* populations from the Senegal River basin and the sudanian population (252 km apart), revealed by our analysis, indicate the existence of continuous populations of this malaria mosquito are inter-connected. Such observations were already reported in the other genetic studies in the populations of *An. funestus*[19,49,51,52] and *An. gambiae s.l*[53-56]. Therefore, these results reassert that the re-colonization of *An. funestus* in the Senegal River basin was probably carried out by a step by step dispersion starting from the neighbouring areas where *An. funestus* had not disappeared.

The genetic analysis clearly distinguished between sahelian and sudanian populations. The genetic tests of isolation by distance suggested that genetic differences observed between domains are linked to the geographical distance. This is a common pattern in *Anopheles*[44,57,58] and particularly in *Anopheles funestus*[18,20,50,51]. In contrast, *An. funestus* populations from the Senegal River basin may be subdivided into two distinct genetic entities: populations around Guiers lake area (Diaminar, Gankette, Loboudou) and populations from the low valley of Senegal River (Mbilor). The existence of these three genetic entities (two in the Senegal River basin and one from the sudanian domain) was confirmed by different genetic approaches (i.e. Structure and AMOVA). When Dielmo was excluded from the isolation by distance analysis, geographical distance did not explain the genetic differentiation observed between Mbilor and populations around Guiers Lake area. Thereby, other factors rather than geographical distance should play a key role into the population structuration of *An. funestus* populations in the Senegal River basin. Chromosomal differentiation detected between *An. funestus* populations of the low valley of Senegal River and the Guiers Lake area (difference of frequency for the inversion 3La) [12] may be implicated in the structure observed as demonstrated in the *An. funestus* populations of Burkina Faso [17]. Because paracentric inversions are involved in the adaptation to various environments, the chromosomal differentiation detected between *An. funestus* populations of the low valley of Senegal River and the Guiers Lake area [12] could be the consequence of different breeding sites, themselves consequences of different environmental changes induced by human activity.

## Conclusions

Our study showed the existence of three genetically different subpopulations of *An. funestus*: populations around Guiers Lake area, populations from the low valley of Senegal River, and populations from Dielmo. The high genetic diversity among populations from the Senegal River basin coupled with no evidence of bottleneck and with a gene flow with the southern population suggested that the re-colonization was likely carried out by massive and repeated stepping-stone dispersions starting from the neighbouring areas where *An. funestus* endured. Geographical distance is not the only factor involved in shaping of the genetic structure observed between the *An. funestus* populations from the low valley of the Senegal River and The Guiers Lake area and we hypothesize that the different breeding sites created by human activities may have shaped chromosomal structuration and may explain the restricted but still occurring gene flow. Our study is therefore indicative of adaptation of malaria vectors to the environment modified by humans.

## Competing interests

The authors declare that they have no competing interests.

## Authors’ contributions

ID, LK and DF designed and supervised the study. BS, ID and LK performed field activities. BS performed laboratory work under guidance of AC. BS, DA, AC analyzed the data. BS, DA, ID and AC drafted and revised the manuscript. All authors approved the final version of the manuscript.

## References

[B1] Coetzee M Fontenille D Advances in the study of *Anopheles funestus*, a major vector of malaria in Africa Insect Biochem Mol Biol 2004 34 599 605 10.1016/j.ibmb.2004.03.012 15242700

[B2] Fontenille D Lepers JP Campbell GH Coluzzi M Rakotoarivony I Coulanges P Malaria transmission and vector biology in Manarintsoa, high plateaux of Madagascar AmJTrop Med Hyg 1990 43 107 115 10.4269/ajtmh.1990.43.107 2202220

[B3] Mendis C Jacobsen J Gamage-Mendis A Bule E Dgedge M Thompson R Cuamba N Barreto J Begtrup K Sinden R Hogh B Anopheles arabiensis and Anopheles funestus are equally important vectors of malaria in Matola coastal suburb of Maputo, southern Mozambique Med Vet Entomol 2000 14 171 180 10.1046/j.1365-2915.2000.00228.x 10872861

[B4] Cohuet A Simard F Wondji CS Antonio-Nkondjio C Awono-Ambene P Fontenille D High malaria transmission intensity due to *Anopheles funestus* (Diptera: Culicidae) in a village of savannah-forest transition area in Cameroon J Med Entomol 2004 41 901 915 10.1603/0022-2585-41.5.901 15535619

[B5] Dabire KR Baldet T Diabate A Dia I Costantini C Cohuet A Guiguemde TR Fontenille D *Anopheles funestus* (Diptera: Culicidae) in a Humid Savannah Area of Western Burkina Faso: Bionomics, Insecticide Resistance Status, and Role in Malaria Transmission J Med Entomol 2007 44 990 997 10.1603/0022-2585(2007)44[990:AFDCIA]2.0.CO;2 18047197

[B6] Diagne N Fontenille D Konate L Faye O Lamizana MT Legros F Molez JF Trape JF Les anophèles du Sénégal. Liste commentée et illustrée Bull Soc Pathol Exot 1994 87 267 277 7866049

[B7] Fontenille D Lochouarn L Diagne N Sokhna CS Lemasson JJ Diatta M Konate L Faye O Rogier C Trape JF High annual and seasonal variations in malaria transmission by anophelines and vector species composition in Dielmo, a holoendemic area in Senegal AmJTrop Med Hyg 1997 56 247 253 10.4269/ajtmh.1997.56.247 9129525

[B8] Dia I Diop T Rakotoarivony I Kengne P Fontenille D Bionomics of *Anopheles gambiae* Giles, *Anopheles arabiensis* Patton, *Anopheles funestus* Giles and *Anopheles nili* (Theobald) (Diptera: Culicidae) and transmission of *Plasmodium falciparum* in a Sudano-Guinean zone (Ngari, Senegal) J Med Entomol 2003 40 279 283 10.1603/0022-2585-40.3.279 12943105

[B9] Dia I Konate L Samb B Sarr J Diop A Rogerie F Faye M Riveau G Remoue F Diallo M Fontenille D Bionomics of malaria vectors and relationship with malaria transmission and epidemiology in three physiographic zones in the Senegal River Basin Acta Trop 2008 105 145 153 10.1016/j.actatropica.2007.10.010 18068685

[B10] Mouchet J Faye O Julvez J Manguin S Drought and malaria retreat in the Sahel West Africa Lancet 1996 348 1736 10.1016/S0140-6736(05)65861-8 8973449

[B11] Konate L Diop A Sy N Faye MN Dieng Y Izri A Faye O Mouchet J Comeback of *Anopheles funestus* in Sahelian Senegal Lancet 2001 358 336 11511072 10.1016/s0140-6736(01)05517-9

[B12] Dia I Samb B Konate L Fontenille D Population structure of newly established *Anopheles funestus* populations in the Senegal River basin using paracentric chromosomal inversions Acta Trop 2010 115 90 94 10.1016/j.actatropica.2010.02.008 20171155

[B13] Boccoloni D Sabatini A Sanogo E Sagnon N’f Coluzzi M Costantini C Chromosomal and vectoriel heterogeneities in Anopheles funestus in Burkina Faso West Africa Parassitologia 1994 36 Supp l1) 20

[B14] Lochouarn L Dia I Boccolini D Coluzzi M Fontenille D Bionomical and cytogenetic heterogeneities of *Anopheles funestus* in Senegal Trans Roy Soc Trop Med Hyg 1998 92 607 612 10.1016/S0035-9203(98)90782-9 10326101

[B15] Costantini C Sagnon N Ilboudo-Sanogo E Coluzzi M Boccolini D Chromosomal and bionomic heterogeneities suggest incipient speciation in *Anopheles funestus* from Burkina Faso Parassitologia 1999 41 595 611 10870569

[B16] Dia I Lochouarn L Boccolini D Costantini C Fontenille D Spatial and temporal variations of the chromosomal inversion polymorphism of *Anopheles funestus* in Senegal Parasite 2000 7 179 183 11031753 10.1051/parasite/2000073179

[B17] Michel AP Guelbeogo WM Grushko O Schemerhorn BJ Kern M Willard MB Sagnon N’f Costantini C Besansky NJ Molecular differentiation between chromosomally defined incipient species of *Anopheles funestus* Insect Mol Biol 2005 14 375 387 10.1111/j.1365-2583.2005.00568.x 16033431

[B18] Ayala D Fontaine CM Cohuet A Fontenille D Vitalis R Simard F Chromosomal Inversions Natural Selection and Adaptation in the Malaria Vector *Anopheles funestus* Mol Biol Evol 2011 28 745 758 10.1093/molbev/msq248 20837604 PMC3002248

[B19] Braginets OP Minakawa N Mbogo CM Yan G Population genetic structure of the African malaria mosquito *Anopheles funestus* in Kenya AmJTrop Med Hyg 2003 69 303 308 14628948

[B20] Cohuet A Dia I Simard F Raymond M Fontenille D Population structure of the malaria vector *Anopheles funestus* in Senegal based on microsatellite and cytogenetic data Insect Mol Biol 2004 13 251 258 10.1111/j.0962-1075.2004.00482.x 15157226

[B21] Gillies MT De Meillon B The Anophelinae of Africa South of the Sahara (Ethiopian zoogeographical region) Publ South Afr Inst Med Res 1968 54 343

[B22] Morlais I Ponçon N Simard F Cohuet A Fontenille D Intraspecific nucleotide variation in *anopheles gambiae*: new insights into the biology of malaria vectors AmJTrop Med Hyg 2004 71 795 802 15642974

[B23] Koekemoer LL Kamau L Hunt RH Coetzee M A cocktail polymerase chain reaction assay to identify members of the *Anopheles funestus* (Diptera: Culicidae) group AmJTrop Med Hyg 2002 66 804 811 10.4269/ajtmh.2002.66.804 12224596

[B24] Cohuet A Simard F Toto JC Kengne P Coetzee M Fontenille D Species identification within the *Anopheles funestus* group of malaria vectors in Cameroon and evidence for a new species AmJTrop Med Hyg 2003 69 200 205 13677376

[B25] Sinkins SP Hackett BJ Costantini C Vulule J Ling YY Collins FH Besansky NJ Isolation of polymorphic microsatellite loci from the malaria vector *Anopheles funestus* Mol Ecol 2000 9 490 492 10.1046/j.1365-294x.2000.00871-2.x 10736053

[B26] Sharakhov I Braginets O Grushko O 12 co-authors A microsatellite map of the African human malaria vector Anopheles funestus J Hered 2004 95 29 34 10.1093/jhered/esh011 14757727

[B27] Cohuet A Simard F Berthomieu A Raymond M Fontenille D Weill M Isolation and characterisation of microsatellite DNA markers in the malaria vector *Anopheles funestus* Mol Ecol Notes 2002 2 498 500 10.1046/j.1471-8286.2002.00290.x

[B28] Bj S Greeman S Banks M Vulule J Sagnon N’f Costantini C Besansky NJ Dinucleotide microsatellite markers from *Anopheles funestus* Mol Ecol Notes 2003 3 505 507 10.1046/j.1471-8286.2003.00493.x

[B29] Belkhir K Borsa P Chikhi L Raufaste N Bonhomme F GENETIX 4.05, logiciel sous Windows TM pour la génétique des populations Laboratoire Génome, Populations, Interactions, CNRS UMR 5000 2004 France: Université de Montpellier II, Montpellier Available from [ http://www.genetix.univ-montp2.fr/genetix/intro.htm

[B30] Goudet J FSTAT version 2.9.3.2. A computer software to calculate F-statistics J Hered 1995 86 485 486

[B31] Raymond M Rousset F GENEPOP, Version 1.2. A population genetics software for exact tests and ecumenicism J Hered 1995 86 248 249

[B32] Wright S Evolution and Genetics of populations Variability within and among natural populations. Volume 4 1978 Chicago: University of Chicago Press

[B33] Weir BS Cockerham CC Estimating F-statistics for the analysis of population structure Evolution 1984 38 1358 1370 10.2307/2408641 28563791

[B34] Goudet J Raymond M De Meeüs T Rousset F Testing differentiation in diploid populations Genetics 1996 144 1933 1940 8978076 10.1093/genetics/144.4.1933 PMC1207740

[B35] Cornuet JM Luikart G Description and power analysis of two tests for detecting recent population bottlenecks from allele frequency data Genetics 1996 144 2001 2014 8978083 10.1093/genetics/144.4.2001 PMC1207747

[B36] Kimura M Ohta T Stepwise mutation model and distribution of allelic frequencies in a finite population Proc Natl Acad Sci USA 1978 75 2868 2872 10.1073/pnas.75.6.2868 275857 PMC392666

[B37] Di Rienzo A Peterson AC Garza JC Valdes AM Slatkin M Freimer NB Mutational processes of simple-sequence repeat loci in human populations Proc Natl Acad Sci USA 1994 91 3166 3170 10.1073/pnas.91.8.3166 8159720 PMC43536

[B38] Pritchard JK Stephens M Donnelly P Inference of population structure using multilocus genotype data Genetics 2000 155 945 959 10835412 10.1093/genetics/155.2.945 PMC1461096

[B39] Falush D Stephens M Pritchard JK Inference of population structure using multilocus genotype data: linked loci and correlated allele frequencies Genetics 2003 164 1567 1587 12930761 10.1093/genetics/164.4.1567 PMC1462648

[B40] Fontaine MC Baird SJE Piry S 19 co-authors Rise of oceanographic barriers in continuous populations of a cetacean: the genetic structure of harbour porpoises in Old World waters BMC Biol 2007 5 30 10.1186/1741-7007-5-30 17651495 PMC1971045

[B41] Excoffier L Lischer HEL Arlequin suite version 3.5: A new series of programs to perform population genetics analyses under Linux and Windows Mol Ecol Resour 2010 10 564 567 10.1111/j.1755-0998.2010.02847.x 21565059

[B42] Rousset F Genetic differentiation and estimation of gene flow from F-statistics under isolation by distance Genetics 1997 145 1219 1228 9093870 10.1093/genetics/145.4.1219 PMC1207888

[B43] Holm S A simple sequentially rejective multiple test procedure Scand J Stat 1979 6 65 70

[B44] Lehmann T Licht M Elissa N Maega BT Chimumbwa JM Watsenga FT Wondji CS Simard F Hawley WA Population Structure of *Anopheles gambiae* in Africa J Hered 2003 94 133 147 10.1093/jhered/esg024 12721225

[B45] Michel AP Grushko O Guelbeogo WM Sagnon N'F Costantini C Besansky NJ Effective population size of Anopheles funestus chromosomal forms in Burkina Faso Malaria J 2006 5 115 10.1186/1475-2875-5-115 PMC1676016 17125511

[B46] Simard F Lehmann T Lemasson JJ Diatta M Fontenille D Persistence of Anopheles arabiensis during the severe dry season conditions in Senegal: an indirect approach using microsatellite loci Insect Mol Biol 2000 9 5 467 479 10.1046/j.1365-2583.2000.00210.x 11029665

[B47] Wondji C Simard F Lehmann T Fondjo E Samè-Ekobo A Fontenille D Impact of insecticide-treated bed nets implementation on the genetic structure of Anopheles arabiensis in an area of irrigated rice fields in the Sahelian region of Cameroon Mol Ecol 2005 14 3683 3693 10.1111/j.1365-294X.2005.02699.x 16202089

[B48] Lenormand T Gene flow and the limits to natural selection Trends Ecol Evol 2002 17 4 183 189 10.1016/S0169-5347(02)02497-7

[B49] Michel AP Ingrasci MJ Schemerhorn BJ Kern M Le Goff G Coetzee M Elissa N Fontenille D Vulule J Lehmann T Sagnon N’f Costantini C Besansky NJ Rangewide population genetic structure of the African malaria vector Anopheles funestus Mo Ecol 2005 14 4235 4248 10.1111/j.1365-294X.2005.02754.x 16313589

[B50] Cohuet A Dia I Simard F Raymond M Rousset F Antonio- Nkondjio C Awono-Ambene PH Wondji CS Fontenille D Gene flow between chromosomal forms of the malaria vector *Anopheles funestus* in Cameroon, Central Africa, and its relevance in malaria fighting Genetics 2005 169 301 311 15677749 10.1534/genetics.103.025031 PMC1448888

[B51] Ayala D Le Goff G Robert V Jong PD Takken W Population structure of the malaria vector *Anopheles funestus* (Diptera : Culicidae) in Madagascar and Comoros Acta Trop 2006 97 292 300 10.1016/j.actatropica.2005.12.002 16464433

[B52] Temu EA Hunt RH Coetzee M Microsatellite DNA polymorphism and heterozygosity in the malaria vector mosquito *Anopheles funestus* (Diptera: Culicidae) in east and southern Africa Acta Trop 2004 90 39 49 10.1016/j.actatropica.2003.10.011 14739021

[B53] Lehmann T Hawley WA Kamau L Fontenille D Simard F Collins FH Genetic differentiation of *Anopheles gambiae* populations from east and west Africa: comparison of microsatellite and allozyme loci Heredity 1996 77 192 200 10.1038/hdy.1996.124 8760401

[B54] Lehmann T Besansky NJ Hawley WA Fahey TG Kamau L Collins FH Microgeographic structure of *Anopheles gambiae* in western Kenya based on mtDNA and microsatellite loci Mol Ecol 1997 6 243 253 10.1046/j.1365-294X.1997.00177.x 9076979

[B55] Chen H Minakawa N Beier J Yan G Population genetic structure of *Anopheles gambiae* mosquitoes on Lake Victoria islands west Kenya Malaria J 2004 3 48 10.1186/1475-2875-3-48 PMC543573 15581429

[B56] Donnelly MJ Townson H Evidence for extensive genetic differentiation among populations of the malaria vector *Anopheles arabiensis* in east Africa Insect Mol Biol 2000 9 357 367 10.1046/j.1365-2583.2000.00197.x 10971713

[B57] Ndo C Antonio-Nkondjio C Cohuet A Ayala D Kengne P Morlais I Awono-Ambene HP Couret D Ngassam P Fontenille D Simard F Population genetic structure of the malaria vector Anopheles nili in sub-Saharan Africa Malar J 2010 9 161 10.1186/1475-2875-9-161 20540796 PMC2898787

[B58] Antonio-Nkondjio C Ndo C Kengne P Mukwaya L Awono-Ambene HP Fontenille D Simard F Population structure of the malaria vector *Anopheles moucheti* in the equatorial forest region of Africa Malar J 2008 7 120 10.1186/1475-2875-7-120 18601716 PMC2483286

